# The *C. elegans* dosage compensation complex mediates interphase X chromosome compaction

**DOI:** 10.1186/1756-8935-7-31

**Published:** 2014-10-27

**Authors:** Alyssa C Lau, Kentaro Nabeshima, Györgyi Csankovszki

**Affiliations:** Department of Molecular, Cellular and Developmental Biology, University of Michigan, Ann Arbor, MI 48109 Michigan; Department of Cell and Developmental Biology, University of Michigan Medical School, Ann Arbor, MI 48109 Michigan

**Keywords:** *Caenorhabditis elegans*, Dosage compensation, Gene expression, Condensin, Chromosome condensation, Chromatin, Interphase chromosome, Epigenetics

## Abstract

**Background:**

Dosage compensation is a specialized gene regulatory mechanism which equalizes X-linked gene expression between sexes. In *Caenorhabditis elegans*, dosage compensation is achieved by the activity of the dosage compensation complex (DCC). The DCC localizes to both X chromosomes in hermaphrodites to downregulate gene expression by half. The DCC contains a subcomplex (condensin I^DC^) similar to the evolutionarily conserved condensin complexes which play fundamental roles in chromosome dynamics during mitosis and meiosis. Therefore, mechanisms related to mitotic chromosome condensation have been long hypothesized to mediate dosage compensation. However experimental evidence was lacking.

**Results:**

Using 3D FISH microscopy to measure the volumes of X and chromosome I territories and to measure distances between individual loci, we show that hermaphrodite worms deficient in DCC proteins have enlarged interphase X chromosomes when compared to wild type. By contrast, chromosome I is unaffected. Interestingly, hermaphrodite worms depleted of condensin I or II show no phenotype. Therefore X chromosome compaction is specific to condensin I^DC^. In addition, we show that SET-1, SET-4, and SIR-2.1, histone modifiers whose activity is regulated by the DCC, need to be present for the compaction of the X chromosome territory.

**Conclusion:**

These results support the idea that condensin I^DC^, and the histone modifications regulated by the DCC, mediate interphase X chromosome compaction. Our results link condensin-mediated chromosome compaction, an activity connected to mitotic chromosome condensation, to chromosome-wide repression of gene expression in interphase.

**Electronic supplementary material:**

The online version of this article (doi:10.1186/1756-8935-7-31) contains supplementary material, which is available to authorized users.

## Background

In many species, such as humans, mice, flies, and worms, sex is determined by a chromosome-based method which entails a difference in sex chromosome number between heterogametic males (XY or XO) and homogametic females (XX). If left uncorrected this difference puts one sex at a disadvantage. Therefore, species have evolved a specialized gene regulatory mechanism to correct this imbalance, known as dosage compensation. Dosage compensation balances X and autosomal expression and equalizes gene expression between the sexes [1].

The molecular mechanism of dosage compensation varies among species. Mammals, flies, and worms achieve dosage compensation using three distinct strategies. In the fly, *Drosophila melanogaster*, the male X is upregulated by two-fold, a mechanism that balances gene expression between the X and autosomes and equalizes male to female X-linked gene expression [2, 3]. In mammals and the nematode *C. elegans*, it is hypothesized that an unknown mechanism upregulates X chromosome expression in both sexes [4–8]. Although X upregulation balances X and autosomal expression in males it also causes X overexpression in females/hermaphrodites. As a result, to prevent hyperexpression of the X chromosomes, mammalian XX females inactivate one X [9–11], while XX hermaphrodite *C. elegans* worms downregulate both X chromosomes two-fold [12, 13]. Although dosage compensation mechanisms vary among species, all lead to the balance between X and autosomal expression and equalize gene expression between the sexes.

In *C. elegans*, dosage compensation is achieved by the dosage compensation complex (DCC) which binds to both X chromosomes to downregulate X-linked gene expression in hermaphrodites. Condensin I^DC^, a subcomplex within the DCC, contains two SMC (structural maintenance of chromosome) proteins (DPY-27 and MIX-1) and three CAP (chromosome-associated polypeptide) proteins (DPY-26, DPY-28, and CAPG-1). In addition, the DCC contains five associated proteins (SDC-1, SDC-2, SDC-3, DPY-30, and DPY-21) [14–21]. Interestingly, condensin I^DC^ differs from the mitotic condensin I complex by only one subunit: DPY-27 replaces SMC-4 [15, 21]. Condensins are evolutionarily conserved complexes, which promote chromosome compaction, organization, and segregation during mitosis and meiosis [22]. Although condensin I^DC^ is homologous to condensin, the DCC appears to have no mitotic function, and it instead functions in the repression of gene expression. Because condensin complexes organize and compact chromosomes in preparation for mitosis, it has been long hypothesized that DCC activity also results in changes in X chromosome compaction [14]. However the direct experimental evidence supporting this hypothesis is lacking.

Condensins (I and II) are conserved protein complexes that organize chromatin structure and whose functions are best studied in mitosis and meiosis. Although structurally similar, the mitotic functions of condensins I and II differ. Condensin I laterally compacts mitotic chromosomes, whereas condensin II mediates axial compaction and rigidity [23–26]. During *C. elegans* meiosis, the depletion of condensin I or condensin II leads to an expansion of chromosome axial length [21]. In *Xenopus laevis* eggs, *S. pombe* and *S. cerevisiae*, condensin is required for mitotic chromosome condensation [27–30], while in other organisms, such as mammals and worms, condensin II is required for prophase condensation [15, 24, 31]. However, in many systems, including *Drosophila*, worms, mammals, and chicken DT40 cells, depletion of condensins I and II leads primarily to defects in anaphase chromosome segregation, such as lagging chromosomes and chromosome bridges, rather than defects in chromosome condensation [22]. Overall, in many organisms, mitotic chromosomes still compact when condensin is disrupted suggesting that condensin is not solely responsible for chromosome condensation.

Condensin activities are well studied in mitosis and meiosis, but the interphase functions of condensins are less understood. In higher eukaryotes condensin I is cytoplasmic in interphase and loads onto chromosomes after nuclear envelope breakdown, whereas condensin II is nuclear throughout the cell cycle [24, 26, 32–34]. Therefore, it is condensin II, rather than condensin I, that is thought to play an important role in the interphase nucleus in these organisms. In *Drosophila*, condensin II activity disrupts somatic homolog pairing and leads to interphase chromosome compaction [35–38]. In addition to compaction, condensin II is required for the proper formation of chromosome territories [39]. Evidence of condensin II-mediated interphase chromosome compaction has also been demonstrated during the development of quiescent naïve T-cells [40] and in mouse embryonic stem cells [41]. In both budding and fission yeast, condensin-dependent RNA polymerase III-transcribed gene clustering at or near the nucleolus contributes to the three-dimensional organization of the genome [42, 43]. Although there is accumulating evidence demonstrating that condensin II, or the single yeast condensin, participates in interphase chromatin organization, whether the condensin I-like complex, condensin I^DC^, organizes the interphase X chromosome in a similar manner is unclear.

Emerging evidence has uncovered that histone tail modifications also influence mitotic chromosome condensation. During cell cycle progression the acetylation of histone H4 lysine 16 (H4K16ac) increases during S phase and decreases during mitosis [44, 45]. By contrast, histone H4 lysine 20 monomethylation (H4K20me1) levels are higher in mitosis [44, 46]. Expression of the H4K20 methyltransferase, PR-SET7, is coincident with the increase of H4K20me1 during mitosis [44, 46]. The negative correlation between these two modifications during the cell cycle is consistent with previous findings that H4K20me1 antagonizes H4K16ac [47]. In yeast, the phosphorylation of histone H3 serine 10 recruits the Sir2 homolog Hst2 to promote deacetylation of H4K16ac. Together, this cascade of histone modifications was proposed to drive chromatin hypercondensation during mitosis, independently from condensin [45].

Interestingly, similar changes in histone modifications are observed on interphase dosage compensated X chromosomes in *C. elegans*. The DCC regulates SET-1 (PR-SET7 homolog) and SET-4 (SUV4-20 homolog), which together mediate the enrichment of H4K20me1 on the X chromosomes [48, 49]. The DCC also regulates SIR-2.1 (Sir2 homolog), which mediates the depletion of H4K16ac on the X chromosomes [49]. These observations suggest that interphase dosage compensated X chromosomes possess some characteristics associated with condensed mitotic chromosome.

In this study, we present experimental evidence linking condensin-mediated chromosome compaction to chromosome-wide repression of gene expression during dosage compensation. We show that hermaphrodite worms deficient in DCC function, as well as male worms, exhibit enlarged X chromosomes when compared to wild type hermaphrodites. This result supports the idea that condensin I^DC^ mediates interphase X chromosome compaction. In addition, we show that DCC-regulated histone modifiers contribute to X chromosome compaction. Together these results give us more insight into the functional link between mitotic chromosome condensation and epigenetic control of gene expression.

## Results

### Dosage compensation mediates changes in X chromosome volumes

**Figure 1:**
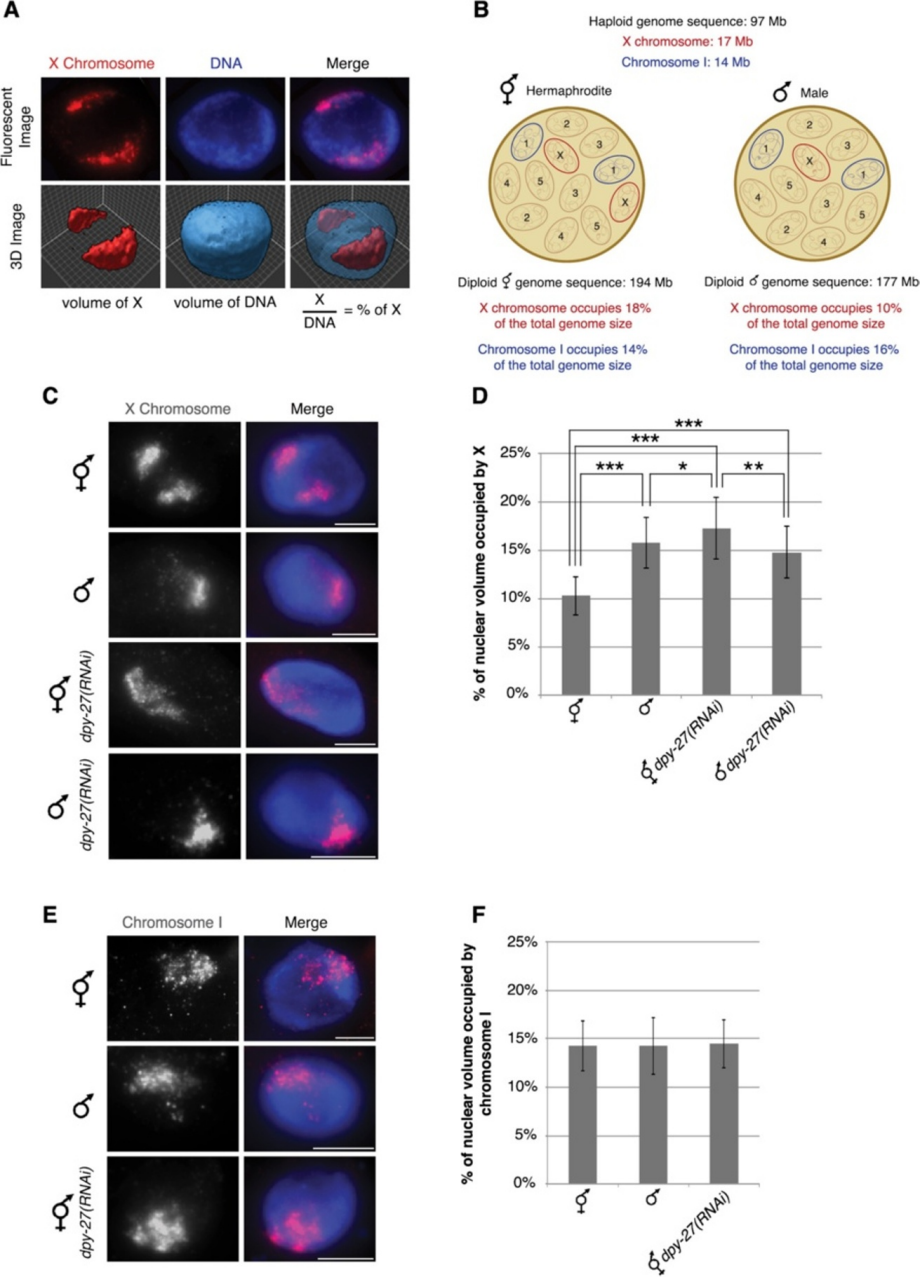
**The absence of the DCC leads to enlarged X territories. (A)** Chromosome-paint 3D FISH measures the volumes of chromosome X and I territories. Representation of how the percentage of nuclear volume occupied by the X or chromosome I paint were quantified in a single nucleus. **(B)** *C. elegans* has five pairs of autosomes and one pair of sex chromosomes. Hermaphrodites have 12 chromosomes with a total genome sequence of 194 Mb. The X chromosomes occupy 18% and chromosome I occupies 14% of the total hermaphrodite genome size. Males have 11 chromosomes with a total genome sequence of 177 Mb. The single X chromosome occupies 10% and chromosome I occupies 16% of the total male genome size. **(C-D)** Adult hermaphrodite and male intestinal nuclei stained with X-paint FISH (red) to label X chromosome territories and DAPI (blue) to label DNA. **(C)** Representative stained nuclei of wild type hermaphrodites, male *him-8(e1489)*, hermaphrodite and male *him-8(e1489) dpy-27* RNAi treated animals. Scale bars equal 5 μm. **(D)** Quantification of the percentage of nuclear volume occupied by X in wild type hermaphrodites (n = 40), male *him-8(e1489)* (n = 40), hermaphrodite *dpy-27(RNAi)* (n = 60), and male *him-8(e1489) dpy-27(RNAi)* (n = 22). Error bars indicate standard deviation. Asterisks indicate level of statistical significance by t-test analysis (one asterisk, *P* <0.05; two asterisks, *P* <0.01; three asterisks, *P* <0.001). **(E, F)** Adult hermaphrodite and male intestinal nuclei stained with chromosome I paint FISH (red) to label chromosome I territories and DAPI (blue) to label DNA. **(E)** Representative stained nuclei of wild type hermaphrodites, male *him-8(e1489)* and hermaphrodite *dpy-27* RNAi treated animals. Scale bars equal 5 μm. **(F)** Quantification of the percentage of nuclear volume occupied by chromosome I in wild type hermaphrodites (n = 40), male *him-8(e1489)* (n = 40) and hermaphrodite *dpy-27(RNAi)* (n = 40). Error bars indicate standard deviation.

To look for changes in chromosome packaging in dosage compensation we utilized chromosome-paint 3D fluorescent *in situ* hybridization (FISH) to measure the volumes of chromosome X and I territories in wild type and DCC-depleted hermaphrodite and male nuclei. These chromosome-paints were generated from yeast artificial chromosome (YAC) DNA and cover approximately 90% of the X chromosome and 86% of chromosome I [50]. We analyzed intestinal nuclei, which are 32-ploid, making visualization and quantification easier [51]. Chromosome territories were defined by selecting intensity threshold based masks of both the chromosome paint signals and the whole nucleus. These defined mask selections allowed us to calculate the volume of the specific chromosome and the whole nucleus. We then quantified the volume of chromosome territories by calculating the percentage of nuclear volume occupied by the X or chromosome I paint in a single nucleus (see Material and Methods) (Figure 1A). Normalization to total nuclear volumes was necessary to minimize the inherent sample-to-sample variation due to the harsh conditions of FISH. Based on DNA content and assuming equal packaging of all chromosomes, the expected percentages for the X and chromosome I are shown on Figure 1B [52]. If DCC activity results in chromosome compaction we expect to see a larger X chromosome territory in DCC-depleted nuclei compared to the wild type hermaphrodites. In addition, because males have one non-dosage compensated X, we would expect the single male X territory to be about half as large as the two non-dosage compensated Xs in DCC-depleted hermaphrodites, but larger than half of the two dosage compensated Xs in wild type hermaphrodites.

We observed that the X chromosome territories in hermaphrodite nuclei depleted of the DCC subunit DPY-27 occupied a significantly larger percentage than in wild type hermaphrodite nuclei (Figure 1C and D). In wild type hermaphrodites the X chromosome territories were compact with a mean percent nuclear volume of 10.31 ± 1.98%. Depletion of the condensin I^DC^-specific protein, DPY-27 [14], by feeding RNAi, led to enlarged X chromosomes compared to wild type, occupying 17.28 ± 3.16% of the nucleus (*P* = 8.49E-22). Interestingly, the volume of the X chromosomes in DPY-27-depleted hermaphrodite nuclei closely correlates with the X chromosome DNA content relative to the total genome size of *C. elegans*, 18%, see Figure 1B. The smaller percent nuclear volume in wild type hermaphrodites suggests that condensin I^DC^ activity results in X chromosomes that are more compact than genomic average.

We predicted that the percent nuclear volume of a single male X would be close to half of the volume of the two DPY-27-depleted hermaphrodite X chromosomes, about 9%, and close to the percent predicted by DNA content, about 10%, see Figure 1B. Surprisingly, we found that the single X chromosome territory in males was larger, occupying the mean percentage of 15.73 ± 2.63. It is possible that the DPY-27-depleted X chromosome territory is not fully decondensed because a small amount of DPY-27 remains after feeding RNAi. An alternate possibility is that chromosome decondensation might contribute to X upregulation in males. X upregulation has been hypothesized to occur in both sexes in mammals and worms to balance the single male X to autosomes; however the mechanism in worms is unknown (see Discussion). We also analyzed males depleted of DPY-27 and found that the X chromosome remained unchanged compared to control males. Together these results support the idea that condensin I^DC^ mediates X chromosome compaction in hermaphrodites and suggest that chromosome decondensation may be involved in X upregulation in males.

We next asked whether the observed enlargement of chromosome territories was specific to the dosage compensated X chromosomes by analyzing the autosomal territory of chromosome I. The percentage occupied by chromosome I was consistent in wild type hermaphrodites (14.26 ± 2.57), males (14.24 ± 2.90), and *dpy-27(RNAi)* hermaphrodite worms (14.46 ± 2.50) (Figure 1E and F). Like the X chromosomes in DPY-27-depleted nuclei, the volume occupied by chromosome I closely correlates with the DNA content of chromosome I relative to the total genome size (Figure 1B). These data indicate that the chromosome territory enlargement in DPY-27-depleted nuclei is not occurring genome-wide but is unique to the dosage compensated X chromosomes.

**Figure 2:**
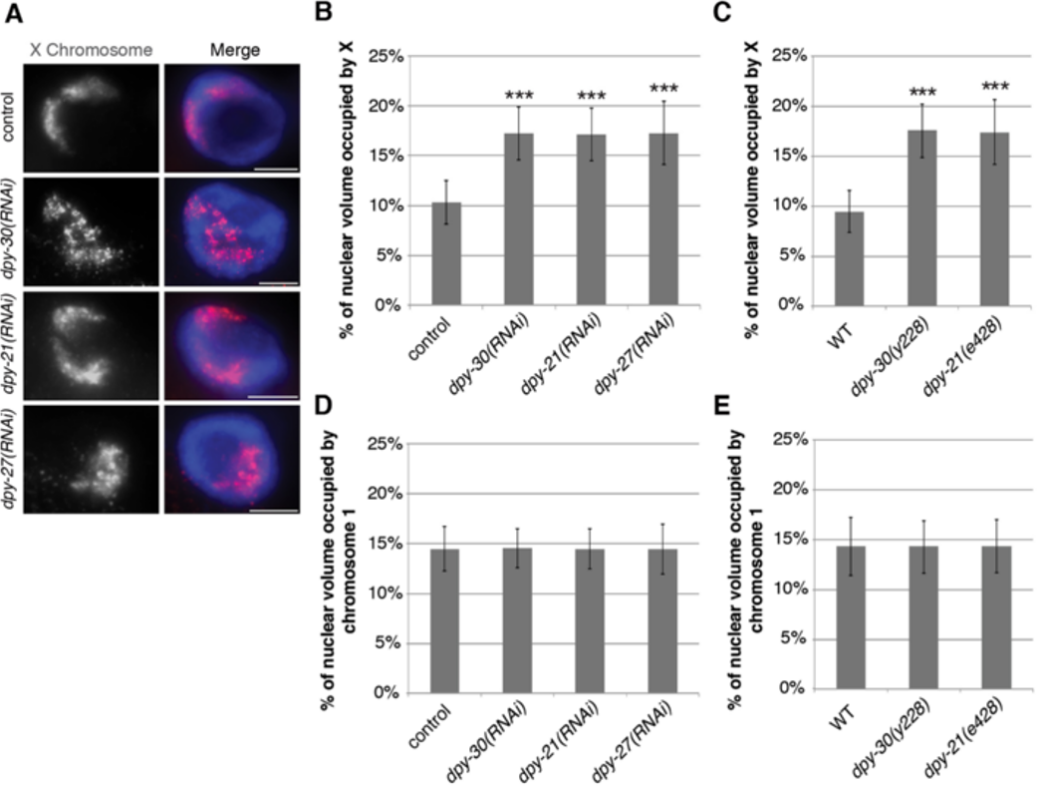
**DCC depletion and mutations disrupt X chromosome compaction. (A, B)** Adult RNAi treated hermaphrodite intestinal nuclei stained with X-paint FISH (red) to label X chromosome territories and DAPI (blue) to label DNA. **(A)** Representative stained nuclei after vector RNAi treatment, *dpy-30(RNAi)*, *dpy-21(RNAi)*, and *dpy-27(RNAi)*. Scale bars equal 5 μm. **(B)** Quantification of the percentage of nuclear volume occupied by X in vector RNAi (n = 60), *dpy-30(RNAi)* (n = 60), *dpy-21(RNAi)* (n = 60), and *dpy-27(RNAi)* (n = 60). Error bars indicate standard deviation. Asterisks indicate level of statistical significance by t-test analysis (three asterisks, *P* <0.001). **(C)** Quantification of the percentage of nuclear volume occupied by X in wild type (n = 36), *dpy-30(y228)* (n = 24), and *dpy-21(e428)* (n = 21). Error bars indicate standard deviation. Asterisks indicate level of statistical significance by t-test analysis (three asterisks, *P* <0.001). **(D, E)** Depletion or mutations of DCC leads to no difference in chromosome I size. **(D)** Quantification of the percentage of nuclear volume occupied by chromosome I in vector RNAi (n = 40), *dpy-30(RNAi)* (n = 40), *dpy-21(RNAi)* (n = 40), and *dpy-27(RNAi)* (n = 40). Error bars indicate standard deviation. **(E)** Quantification of the percentage of nuclear volume occupied by chromosome I in wild type (n = 25), *dpy-30(y228)* (n = 25), and *dpy-21(e428)* (n = 25). Error bars indicate standard deviation.

Next, we wanted to test whether the loss of DCC subunits other than DPY-27 has similar effects on X chromosome volume. We performed the same analysis as described above in worms depleted of, or carrying mutations in the genes encoding, either DPY-30 or DPY-21. We chose these specific genes because dosage compensation function and DCC localization is disrupted when DPY-30 is depleted [53, 54], whereas in DPY-21 depleted worms dosage compensation function is disrupted but the DCC still localizes to the X chromosome [20]. Similar to *dpy-27(RNAi)* worms, *dpy-30(RNAi)* and *dpy-21(RNAi)* worms as well as *dpy-30(y228)* and *dpy-21(e428)* mutants had larger X chromosomes compared to control worms fed bacteria carrying an empty vector or wild type worms (Figure 2A-C and Additional file 1: Figure S1). The mean percentages occupied by the X chromosomes in the DCC-depleted animals were 70% larger than the control. In addition, the size of chromosome I territory was unchanged, occupying a mean percentage of 14.5 in all backgrounds (Figure 2D and E, and Additional file 1: Figure S1). Taken together, these results indicate that wild type and control intestinal nuclei have compact X chromosome territories and this organizational pattern is dependent upon dosage compensation.

Consistent with dosage compensation’s role in reducing gene expression, our data show that the relative size of X chromosomes is also reduced. We next wanted to determine if the two X chromosomes behave similarly and are compacted to similar extents. We performed this analysis in the 32-ploid intestinal nuclei. We measured individual chromosome volumes, arbitrarily designating the larger territory X_1_ and the smaller territory X_2_. Fifty-two percent of vector control worms, and 33% of *dpy-27(RNAi)* worms had two clear X chromosome territories. We observed that both chromosome territories X_1_ and X_2_ in hermaphrodite nuclei depleted of DPY-27 occupied a significantly larger percentage than in vector control nuclei (Additional file 1: Figure S1). The caveat of this experiment is that the chromosomes are not individually marked and the X_1_ territory in one genotype does not necessarily corresponds to X_1_ in the other. However, together with the observation that both X chromosomes associate with the dosage compensation complex, we favor the interpretation that both Xs are affected to similar degrees.

### Diploid DCC-depleted nuclei also have enlarged X chromosomes

**Figure 3:**
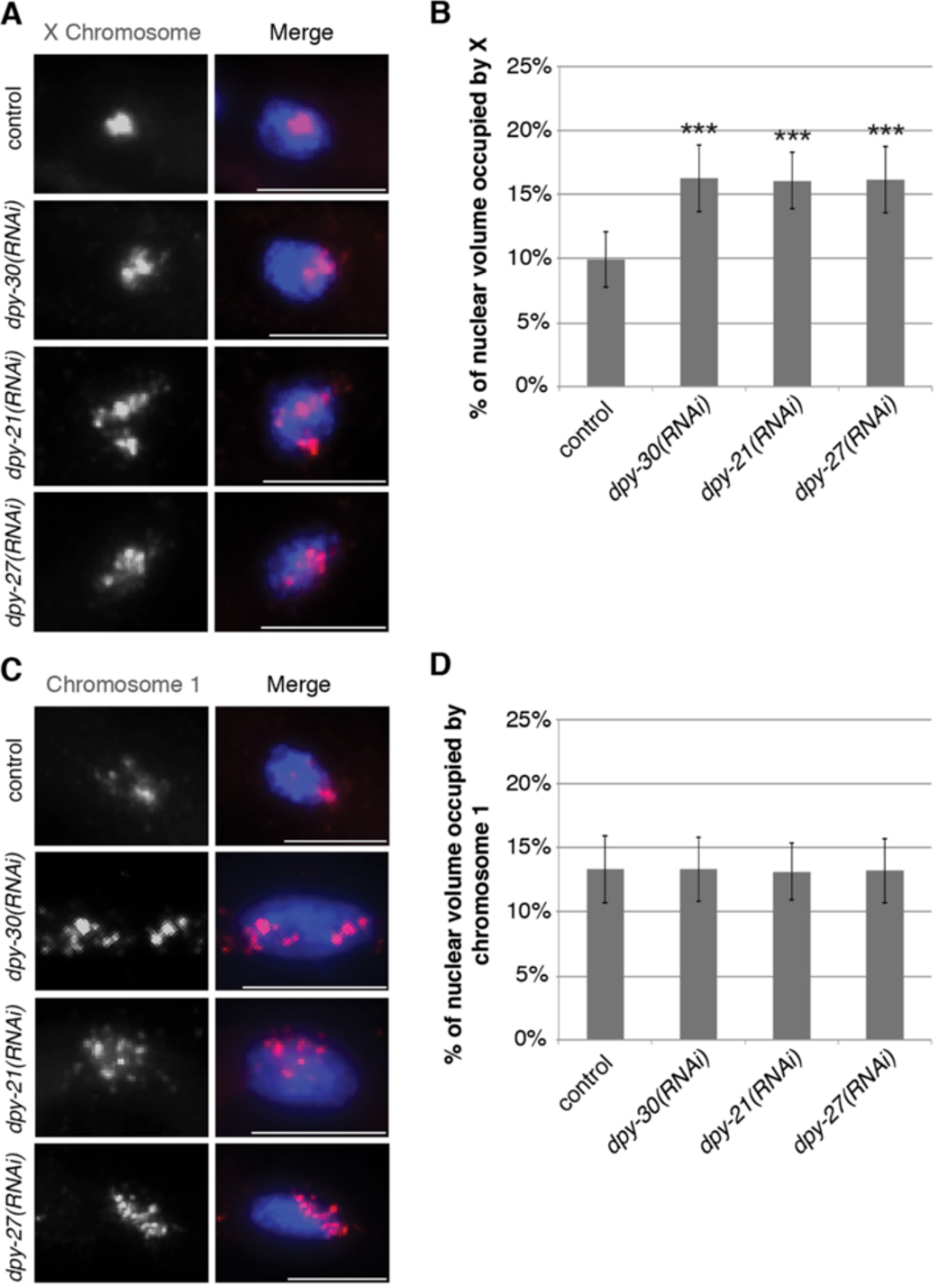
**The decondensed X chromatin structure in DCC-depleted worms is a result of defective compaction. (A, B)** Adult RNAi treated hermaphrodite diploid nuclei stained with X-paint FISH (red) to label X chromosome territories and DAPI (blue) to label DNA. **(A)** Representative stained nuclei after vector RNAi treatment, *dpy-30(RNAi)*, *dpy-21(RNAi)*, and *dpy-27(RNAi)*. Scale bars equal 5 μm. **(B)** Quantification of the percentage of nuclear volume occupied by X in vector RNAi (n = 40), *dpy-30(RNAi)* (n = 40), *dpy-21(RNAi)* (n = 40), and *dpy-27(RNAi)* (n = 40). Error bars indicate standard deviation. Asterisks indicate level of statistical significance by t-test analysis (three asterisks, *P* <0.001). **(C, D)** Adult hermaphrodite diploid nuclei stained with chromosome I paint FISH (red) to label chromosome I territories and DAPI (blue) to label DNA after DCC depletion. **(C)** Representative stained nuclei after vector RNAi treatment, *dpy-30(RNAi)*, *dpy-21(RNAi)*, and *dpy-27(RNAi)*. Scale bars equal 5 μm. **(D)** Quantification of the percentage of nuclear volume occupied by chromosome I in vector RNAi (n = 40), *dpy-30(RNAi)* (n = 40), *dpy-21(RNAi)* (n = 40), and *dpy-27(RNAi)* (n = 40). Error bars indicate standard deviation.

Because intestinal nuclei are 32-ploid, the possibility remained that the enlarged X territories were a result of the dispersing of the multiple copies of the X chromosomes and not a result of perturbed compaction of individual chromosomes. To test this possibility, we examined the X chromosomes in the diploid tail tip hypodermal cells, hyp 8-11 [55]. We found similar results to that of intestinal nuclei in the diploid cells (Figure 3A and B). The X chromosome territories in vector control RNAi diploid cells were tightly compact occupying 9.94 ± 2.20%, whereas the X chromosome territories in *dpy-30*, *dpy-2*1, and *dpy-27* RNAi diploid cells were decondensed occupying 16.27 ± 2.62% (*P* = 6.70E-19), 16.09 ± 2.22% (*P* = 3.02E-20), and 16.17 ± 2.59% (*P* = 1.15E-18), respectively. Additionally, the percent volume occupied by chromosome I in diploid cells was not statistically different amongst the control and DCC-depleted animals, averaging 13.2% in all cases (Figure 3C and D). This suggests that the decondensed X chromatin structure in DCC-depleted worms is not solely a result of the dispersing of the multiple copies of the X chromosome in the 32-ploid intestinal nuclei, but it is a result of defective compaction. We did not look at separate X chromosome territories in diploid cells because due to their small size only a small percentage of nuclei had clearly distinguishable X chromosome territories. Only 35% of both vector and *dpy-27(RNAi)* diploid nuclei clearly exhibited two separate X chromosome territories.

### X chromatin compaction is evident at all genomic distances examined

**Figure 4:**
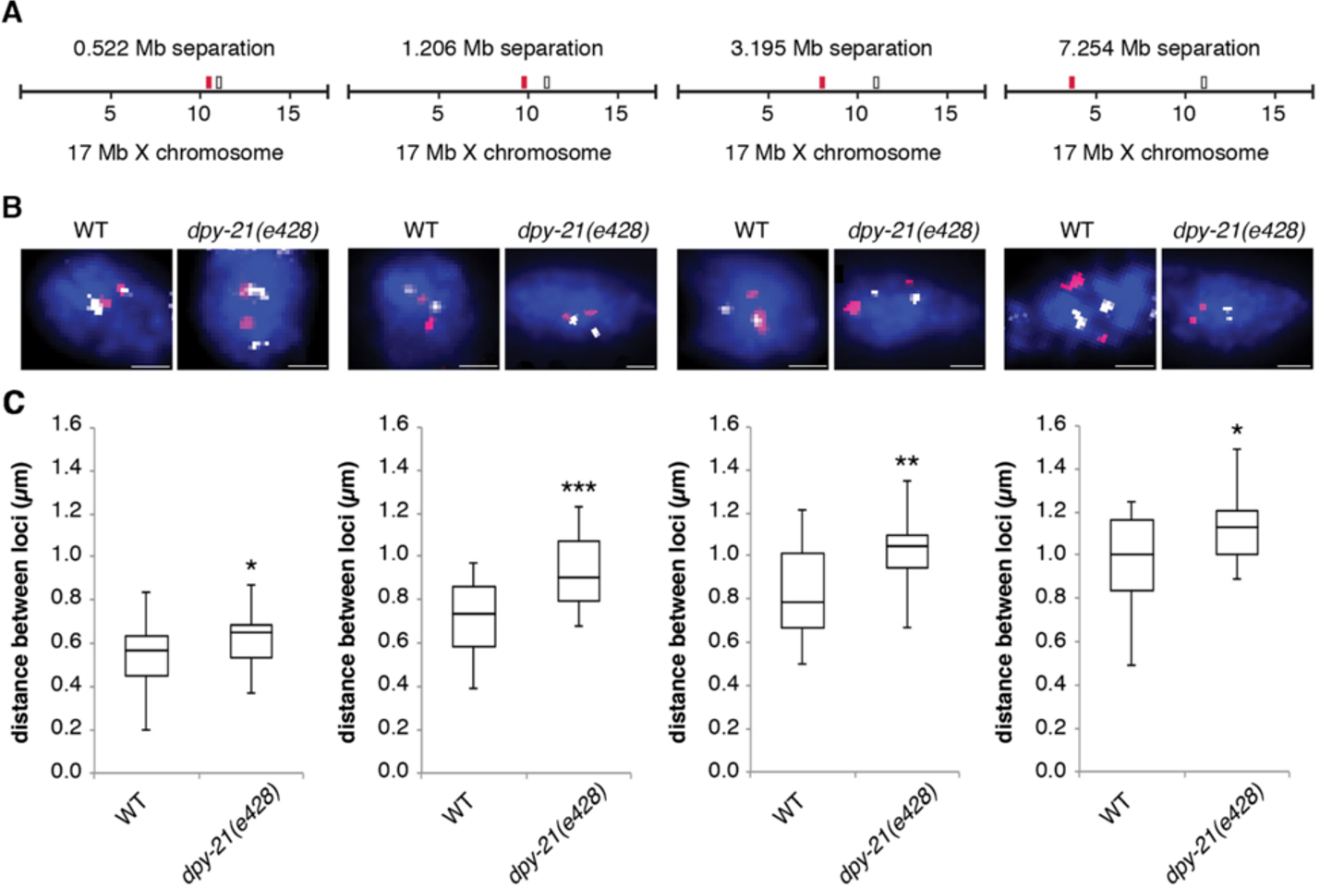
**X chromatin compaction is evident at all genomic distances examined. (A)** FISH probe pairs across the X chromosome. The position of YAC probes (red and white boxes) used in FISH is indicated. **(B)** 2D projections of 3D stacked images. Representative stained diploid nuclei of adult hermaphrodite wild type and *dpy-21(e428)* worms. Nuclei stained with probes pairs across the X chromosome (red and white) and counterstained with DAPI (blue) to label DNA. Scale bars equal 1 μm. **(C)** Boxplots indicating the distribution of 3D loci distances for wild type (n = 20) and *dpy-21(e428)* (n = 20) diploid nuclei. Boxes show the median and interquartile range of the data. Asterisks indicate level of statistical significance by t-test analysis (one asterisk, *P* <0.05; two asterisks, *P* <0.01; three asterisks, *P* <0.001).

To further investigate the genomic scale at which condensin operates we performed 3D FISH with pairs of X chromosome YAC probes separated by genomic distances ranging from 0.5 Mb to 7.2 Mb (Figure 4A and B). Such analysis has been previously used to demonstrate a role for polycomb repressive complexes in compacting chromatin in mouse embryonic stem cells, and a role for condensin II to promote compaction of chromosome territories in *Drosophila* [39, 56]. Since this analysis is not possible in polyploidy intestinal nuclei we analyzed the pairs of probes in wild type and *dpy-21(e428)* mutant diploid tail tip cells. Eighty-three percent of wild type diploid cells and 79% of *dpy-21(e428)* diploid cells had two clear spots for each probe, while others had either no spots due to high background or had one spot (presumably due to the overlap of two closely spaced spots). Nuclei with 0 or one spot were excluded from our analysis. No nuclei had three or four spots. These observations indicate that these cells have an unreplicated diploid DNA content. At all four genomic distances we detected a significant increase in distances in *dpy-21(e428)* mutants compared to wild type (Figure 4C). This more dispersed distribution of the two loci found in *dpy-21(e428)* correlates with the larger X chromosome territories found in the dosage compensation mutants. These data suggest that dosage compensation can be linked to levels of higher-order X chromatin compaction, both at the level of whole chromosomes and at a genomic scale as small as 0.5 Mb and as large as 7.2 Mb.

### X chromosome compaction is not regulated by condensin I or condensin II

**Figure 5:**
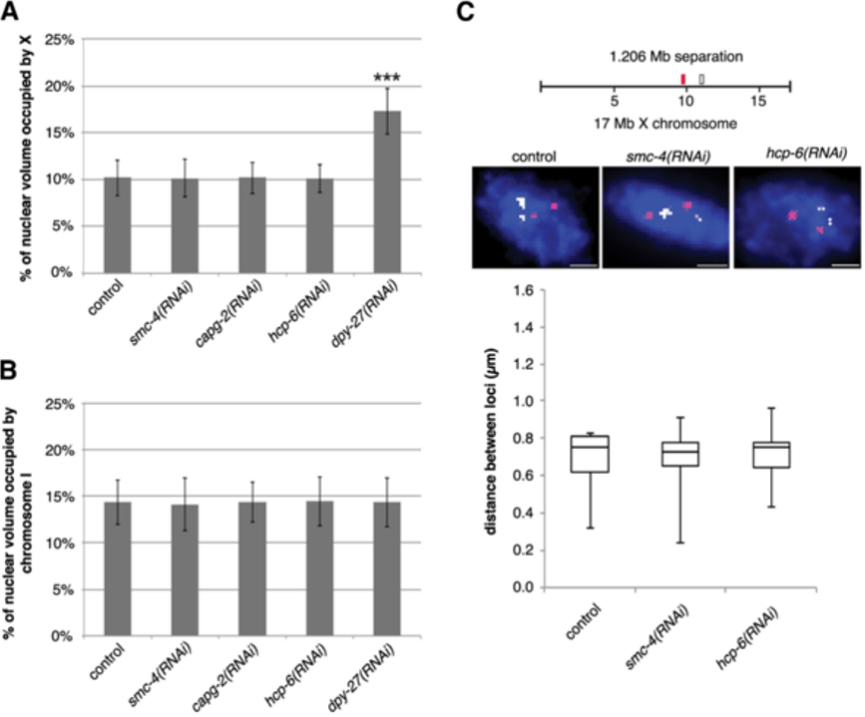
**X chromosome compaction is not disrupted in condensin I or II depleted animals. (A)** Quantification of the percentage of nuclear volume occupied by X in vector RNAi (n = 40), *smc-4(RNAi)* (n = 28), *capg-2(RNAi)* (n = 40), *hcp-6(RNAi)* (n = 40), and *dpy-27(RNAi)* (n = 34). Error bars indicate standard deviation. Asterisks indicate level of statistical significance by t-test analysis (three asterisks, *P* <0.001). **(B)** Quantification of the percentage of nuclear volume occupied by chromosome I in vector RNAi (n = 40), *smc-4(RNAi)* (n = 29), *capg-2(RNAi)* (n = 40), *hcp-6(RNAi)* (n = 40), and *dpy-27(RNAi)* (n = 32). Error bars indicate standard deviation. **(C)** FISH probe pairs across the X chromosome. The position of YAC probes (red and white boxes) used in FISH is indicated. 2D projections of 3D stacked images. Representative stained diploid nuclei of vector RNAi, *smc-4(RNAi)* and *hcp-6(RNAi)* worms. Nuclei stained with probes pairs across the X chromosome (red and white) and counterstained with DAPI (blue) to label DNA. Scale bars equal 1 μm. Boxplots indicating the distribution of 3D loci distances for vector RNAi (n = 20) and *smc-4(RNAi)* (n = 20) and *hcp-6(RNAi)* (n = 20) diploid nuclei. Boxes show the median and interquartile range of the data.

Previous studies have found that condensin II promotes the formation of interphase chromosome territories in *Drosophila* [35, 39]. Therefore, we next wanted to investigate whether the X chromosome compaction was specific to condensin I^DC^ or if condensin I or II are also contributing to this phenotype. To test this, we depleted SMC-4, CAPG-2, or HCP-6. SMC-4 is a subunit of both condensins I and II, while CAPG-2 and HCP-6 are subunits specific to condensin II [15, 21, 31, 57, 58]. We performed a shorter, one generation RNAi feeding of SMC-4, CAPG-2, HCP-6, DPY-27, and empty vector, due to the lethality of SMC-4, CAPG-2, or HCP-6 depletion over two generations. One generation RNAi feeding depleted condensin subunits to below level of detection by western blotting (Additional file 2: Figure S2). In addition the presence of chromatin bridges between many nuclei, a hallmark of chromosome segregation defects, in SMC-4, CAPG-2, and HCP-6-depleted worms, indicated successful depletion. Depleting SMC-4, CAPG-2, or HCP-6 did not change the level of compaction compared to control vector RNAi worms (Figure 5A and Additional file 2: Figure S2). The mean volume occupied by the X chromosomes was consistently around 10.1%. However, even with the shorter one generation RNAi depletion, *dpy-27(RNAi)* X chromosome territories were large at 17.29 ± 2.45%. Similarly, there was no change in the volume of chromosome I when either SMC-4, CAPG-2, or HCP-6 were depleted compared to control animals (Figure 5B and Additional file 2: Figure S2). Additionally, the same analysis was performed on the diploid tail tip hypodermal cells and similar results were found (Additional file 3: Figure S3). Similar conclusions were reached when using 3D FISH with pairs of X chromosome YAC probes separated by the genomic distance of 1.2 Mb, the distance that showed the most significant difference between *dpy-21(e428)* mutants and wild type. 83% of *smc-4(RNAi)* diploid nuclei and 85% of *hcp-6(RNAi)* diploid nuclei had two clear spots for each probe, while no nuclei had three or four spots. At the genomic distance of 1.2 MB we did not detect a significant change in distances in *smc-4(RNAi)* or *hcp-6(RNAi)* worms compared to vector control worms (Figure 5C). Therefore, we conclude that condensin I^DC^, and not condensin I or condensin II, is primarily responsible for dosage compensation mediated X chromosome compaction. However we cannot rule out the possibility that condensin I or II are affecting interphase chromosome territories in *C. elegans* at levels undetectable by 3D FISH.

### Changes in DCC-mediated histone modifiers effect X chromosome structure

**Figure 6:**
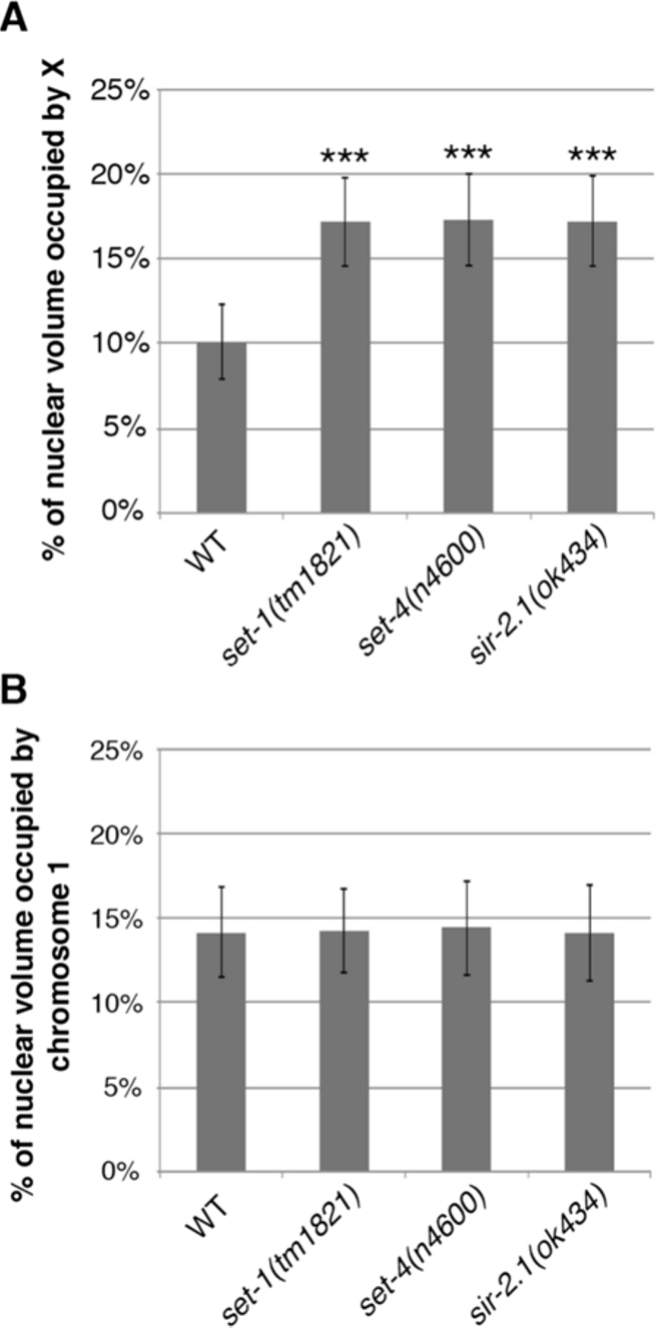
**Depletion of the DCC-meditated histone modifiers leads to the loss of X chromosome compaction. (A)** Quantification of the percentage of nuclear volume occupied by X in wild type (n = 40), *set-1(tm1821)* (n = 40), *set-4(n4600)* (n = 40) and *sir-2.1(ok434)* (n = 40). Error bars indicate standard deviation. Asterisks indicate level of statistical significance by t-test analysis (three asterisks, *P* <0.001). **(B)** Quantification of the percentage of nuclear volume occupied by chromosome I in wild type (n = 40), *set-1(tm1821)* (n = 20), *set-4(n4600)* (n = 40), and *sir-2.1(ok434)* (n = 40). Error bars indicate standard deviation.

We, and others, previously showed that DCC activity leads to the enrichment of H4K20me1 on the X chromosome by the methyltransferases SET-1 and SET-4 [48, 49]. This activity then leads to the depletion of H4K16ac levels on X, via the deacetylase SIR-2.1 [49]. In order to determine whether these chromatin modifications contribute to compaction of the X, we examined *set-1(tm1821)*, *set-4(n4600)*, and *sir-2.1(ok434)* mutant worms. Mutations in *set-1*, *set-4*, or *sir-2.1* led to the loss of X chromosome compaction seen in wild type worms (Figure 6A and Additional file 4: Figure S4). However, chromosome I showed no significant change in volume in these histone modifier mutants (Figure 6B and Additional file 4: Figure S4). Interestingly, the DCC localizes normally to the X in *set-1(tm1821)*, *set-4(n4600)*, and *sir-2.1(ok434)* worms [48, 49]. Therefore, DCC alone is not sufficient for the compaction of the X chromosome territory. We conclude that X chromosome compaction by the DCC requires the presence of these chromatin modifiers.

## Discussion

**Figure 7:**
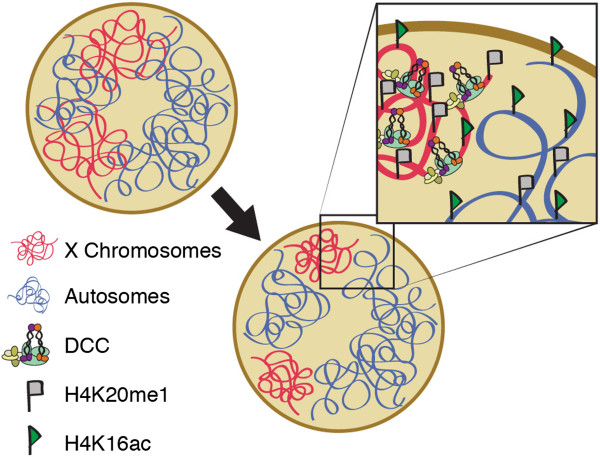
**X chromosome compaction is dependent on the DCC and the DCC-mediated histone modifications.** A graphical cartoon illustrates DCC and DCC-mediated histone modifications effects on hermaphrodite X chromosome structure. The DCC binds to both hermaphrodite X chromosomes and by regulating SET-1 and SET-4, mediates an enrichment of H4K20me on the X chromosome, which, in turn, through SIR-2.1 activity, depletes H4K16ac. These activities compact the X chromosome territories and downregulate X-linked gene expression by half.

In this study, we sought to determine whether the condensin-like DCC compacts chromosomes for gene expression. We established that in wild type hermaphrodites, the interphase X chromosomes are more compact than predicted based on DNA content. This compaction is dependent on the presence of the DCC as well as the DCC-regulated histone modifiers SET-1, SET-4, and SIR-2.1 (Figure 7). Together, our results suggest that mechanisms related to mitotic chromosome condensation mediate dosage compensation.

Previous gene expression studies in DCC mutants showed that X expression increases while autosomal expression decreases compared to wild type [59]. Assuming that these gene expression changes are reflected in chromosome volume changes, we would predict that an increase in the volume of the X is accompanied by a decrease in the volume of autosomes. If the X is occupying an extra 7% of the nucleus, the five pairs of autosomes together will occupy 7% less, approximately 1.4% less for each autosome. We did not detect any decrease in chromosome I volume, but our method may not be sensitive enough to detect such a small change.

### Condensins and cell cycle regulation

Condensins are conserved complexes that play fundamental roles in chromosome dynamics throughout the cell cycle [22]. Similar to conserved condensin II activities, *C. elegans* condensin II is required for prophase chromosome condensation and anaphase segregation [31, 58]. However, condensin I’s role in *C. elegans* is less understood. It is believed that condensin I plays a less critical role in mitosis and development than condensin II, because condensin I depletions lead to less severe phenotypes [15]. Condensin’s role in mitosis and meiosis has been widely studied, however recent evidence revealed that the condensin complexes also contribute to a variety of interphase chromosome functions. A prominent example is the *C. elegans* condensin I-like complex, condensin I^DC^, which does not function in mitosis or meiosis, but instead regulates chromosome- and sex-specific gene expression [12]. Interestingly, condensin I^DC^ differs from the condensin I complex by only one subunit. It is assumed that during evolution, the condensin I subunit SMC-4 duplicated and diverged giving rise to DPY-27. This resulted in the formation of condensin I^DC^ with the specialized function of dosage compensation. It is believed that the mechanism of dosage compensation is closely linked to condensin’s role in regulating chromosome architecture.

In this study, we report that condensin I^DC^ is responsible for compacting interphase X chromosome territory, both at the level of whole chromosome territories, and by measuring 3D-distances between probe pairs separated by various genomic distances. Evidence of condensin-mediated interphase chromosome compaction has also been found in *Drosophila* and mice [35, 39–41]. Condensin activity is required to unpair polytene chromosomes and disperse heterochromatin in *Drosophila* [39]. In mice, the condensin-mediated condensation is required for proper T-cell development and maintenance of the quiescent state [40]. Condensin has also been found to be required for chromatin compaction and viability in ES cell [41]. In these systems, condensin mediates chromosome changes genome-wide. By contrast, in *C. elegans*, condensin I^DC^ causes chromosome specific changes. It is also interesting to note that in flies and mice condensin II plays a role in interphase chromosome [35, 39–41], while our data do not support a role for condensin II, only condensin I^DC^. These differences between our findings and previous data may reflect a difference between the species or the type of cells examined. Indeed, cell-type specific differences in condensin usage have been observed before, at least in the context of mitotic chromosome condensation [25].

It should be noted that our studies were performed on postmitotic cells. Whether there are differences in how condensin I^DC^ affects X chromosome compaction in G1, S, or G2 phase of cycling cells is unknown. However, a chromatin mark associated with dosage compensation, enrichment of H4K20me1, appears several cell cycles after the DCC assembles on the X chromosomes [48, 60], suggesting that full compaction may require passage through mitosis.

### Molecular mechanisms of condensin

Although some of the biological functions of condensins have been uncovered, our understanding of condensin’s molecular mechanisms remains poor. Condensins contain two SMC (structural maintenance of chromosomes) proteins and three CAP (chromosome-associated polypeptide) proteins. The two SMC proteins are ATPases, and their ability to hydrolyze ATP is essential for condensin function [22]. One of the better-studied biochemical activities of condensin, found in many eukaryotic species, is its ability to supercoil DNA *in vitro*. This supercoiling requires its ATPase activity, as well as all five subunits [31, 61–64]. The introduction of positive supercoiling is proposed to lead to the formation of chiral loops [22, 65] and facilitate decatenation of sister chromatids by topoisomerase II [66]. This would further compact the chromatin fibers to form higher order assemblies. In addition to supercoiling, the SMC proteins of condensins have been found to play a role in reannealing complementary ssDNAs into dsDNAs [67]. Condensins’ reannealing activity might help ‘fix’ ssDNA to prepare it for the formation of mitotic chromosomes. Condensin is also believed to entrap the chromatin fiber by encircling two distinct segments of the chromatin fiber [68]. Together these ATP-dependent biochemical activities are thought to result in condensed mitotic chromosomes. In *C. elegans* specific point mutations of the ATP-binding motifs of DPY-27 and MIX-1 disrupt dosage compensation [14, 16]. These results suggest another link between condensin’s function in mitosis and dosage compensation.

### Condensin and chromatin regulated chromosome compaction

In addition to condensin-mediated mitotic chromosome condensation, histone modifications have also been found to contribute to chromatin hypercondensation during mitosis [44–46]. Interestingly, similar histone modifications are present on interphase dosage compensated X chromosomes in *C. elegans*. The monomethylation of H4K20 is increased, whereas acetylation of H4K16 is decreased, both on mitotic chromosomes and on interphase dosage compensated X chromosomes [44–46, 48, 49]. This is consistent with the hypothesis that mechanisms related to mitotic chromosome condensation mediate dosage compensation. In mitosis, H4K16 deacetylation leads to a stronger interaction between H2A and H4 on adjacent nucleosomes, thus leading to the formation of higher order chromosomes packaging [45, 69]. Our results suggest that DCC-mediated X chromosome compaction requires both the enrichment of H4K20me1 and the depletion of H4K16ac on the X chromosomes similar to the events occurring in mitosis. However, we cannot rule out the possibility that additional factors also contribute to the compaction of the X chromosome. In mitosis, it is believed that this cascade of histone modifications is acting independently of condensin to compact chromosomes [45]. By contrast, X-enrichment of H4K20me1 and depletion of H4K16ac during dosage compensation is DCC- (therefore condensin-) dependent [49]. It will be interesting to determine the exact mechanistic contributions of these modifications both to mitotic chromosome condensation and dosage compensation.

### Upregulation of the X chromosome

X upregulation balances the single male X to autosomes. In flies, X upregulation is male specific, but in mammals and worms it is hypothesized that upregulation occurs in both sexes. Male-specific upregulation of the X chromosome in *Drosophila* is mediated by the MSL (male-specific lethal) complex [70, 71]. The MSL complex binds to the male X chromosome, concentrates MOF acetyltransferase activity, and leads to increased H4K16ac on the hyperactive X [72, 73]. The MSL complex has been shown to mediate two chromatin alterations. First, the presence of the complex reduces the level of negative supercoiling [74]. Second, the acetylation of H4K16 alone weakens nucleosome packing in a chromatin fiber and causes chromatin decondensation on the male X chromosome [75]. Microarray and RNA-seq analysis have provided some evidence for X upregulation in mammals and worms [4–8]. In mammals it is believed that H4K16ac and enhanced transcription initiation contribute to X upregulation [4]. However, the mechanism in worms is unknown, but it may involve changes in chromatin structure. Our data show that in *C. elegans* the single male X chromosome territory is larger than expected. This result suggests that chromosome decondensation might contribute to the X upregulation mechanism. It will be of great interest to explore what is mediating chromosome decondensation in *C. elegans* males. Previous studies have indicated that in the absence of DCC activity, H4K16ac is enriched on hermaphrodite X chromosomes, suggesting that this mark may be involved in the X upregulation process. However, males show no enrichment of H4K16ac by immunofluorescence microscopy [49]. In addition, there is no enrichment of H4K16ac on the X chromosomes in early hermaphrodite, male, or DCC-mutant embryos [60]. Therefore, further investigation is required to determine the mechanisms causing chromosome decondensation and X upregulation in males.

## Conclusions

It has been long hypothesized that DCC activity results in interphase X chromosome compaction. However, the experimental evidence was lacking. Our results are consistent with this long standing hypothesis suggesting that dosage compensated X chromosomes maintain some characteristics associated with condensed mitotic chromosome. Our studies of *C. elegans* condensin I^DC^-mediated compaction may shed further light on the mechanisms of chromatin organization and gene repression by condensin in other organisms. Future studies will examine the factors that directly contribute to X upregulation and possibly chromosome decondensation in males.

## Methods

### Strains

All experimental procedures on *C. elegans* followed internationally recognized guidelines and were approved by the University of Michigan Institutional Biosafety Committee, which follows guidelines established by the National Institute of Health. IBC registration number: IBC00000599. All strains were maintained on NG agar plates with *E. coli* (OP50) as a food source, using standard methods [76]. Strains include: N2 Bristol strain (wild type); TY4403 *him-8(e1489)* IV; TY1936 *dpy-30(y228)* V/nT1 (*unc-?* (n754*) let-?*); TY3936 *dpy-21(e428)*; SS1075 *set-1(tm1821)* III/ hT2g; MT14911 *set-4(n4600)* II; VC199 *sir-2.1(ok434)* IV. Males were obtained from *him-8(e1489)* hermaphrodites. Mutations in *him-8* cause X chromosome nondisjunction in meiosis and result in 38% of progeny being XO males.

### RNA interference

*E. coli* HT115 bacteria expressing double stranded RNA for *dpy-30*, *dpy-21*, *dpy-27*, *smc-4*, *capg-2*, *hcp-6*, or vector control (polylinker), were used for feeding RNAi using the Ahringer laboratory RNAi feeding library [77]. One generation feeding RNAi (WT on *smc-4* RNAi, WT on *capg-2* RNAi, WT on *hcp-6* RNAi) was performed as follows: L1-stage larvae were placed on plates seeded with RNAi bacteria and grown to adulthood. Two generation feeding RNAi (all other analysis) was performed as follows; P_0_ adults from one generation feeding RNAi were transferred to new RNAi plates to produce progeny for 24 h. These progeny (F_1_ generation) were grown to adulthood and examined.

### Fluorescent *in situ*hybridization (FISH)

FISH probe templates were generated by degenerate oligonucleotide primed PCR to amplify purified yeast artificial chromosome (YAC) DNA [50, 78]. The labeled chromosome-paint probes were prepared as described previously [78]. To perform FISH, adult animals (24 h post-L4) with or without previous RNAi treatment were dissected and fixed in 2% PFA, 1× sperm salts, on a slide for 5 min at room temperature. The slide was covered with a coverslip and placed on a dry ice block for 10 min. The coverslip was quickly removed from the slide, and the slides were washed three times in PBST for 10 min each, dehydrated through an ethanol series (70%, 80%, 95%, 100% ethanol, 2 min each) and air dried. A total of 10 uL of probe was added to the slide. The samples were covered with a coverslip, placed on a 95°C heat block for 3 min, and slowly cooled to 37°C for an overnight incubation. The following washing regime was used at 37°C: three 5-min washes in 2× SSC with 50% formamide, then three 5-min washes in 2× SSC, and one 10-min wash in 1× SSC. Samples were incubated in PBST for 10 minutes with DAPI. Slides were mounted with Vectashield (Vector Laboratories) [78].

### Microscopy and image analysis

Images were captured with a Hamamatsu Orca-Erga close-coupled-device (CCD) camera mounted on an Olympus BX61 motorized Z-drive microscope using a 60× APO oil immersion objective. 3D image stacks were collected for each nucleus at 0.2 micrometer Z-spacing. Projection images were generated from these optical sections.

For volume measurements, masks were set using the ‘mask → segment’ function. The mask is established by a user-defined intensity threshold value applied over an image in order to distinguish real signal from background signal and autofluorescence. The same standard of background signal exclusion was applied to all nuclei, based upon the levels of background signal and autofluorescence observed. This was done for each channel of an image. The total volume of the ‘whole nucleus’ mask was calculated from DAPI signal. DAPI signal was set as the primary mask and the three dimensional pixels (voxels) were measured through Slidebook (morphometry → volume). The volume of the X chromosome or chromosome I mask was calculated from the paint signals. Chromosome-paint signals were set as the secondary mask and for each object in the primary mask. The number of overlapping voxels in the secondary mask was calculated by Slidebook (cross mask → mask overlaps). The percentage of nuclear volume occupied by either the X chromosome or chromosome I was obtained by dividing the volume of the specific chromosome over the volume of the whole nucleus. This percentage was calculated for each nucleus within an experimental set. The percentages where then averaged over all nuclei within an experimental set to calculate the final mean percentage of nuclear volume occupied by the X chromosome or chromosome I value shown on each graph. Descriptive statistics (standard deviation and sample size) were also calculated. Sample sizes are listed in each figure. Error bars shown are means +/- 1 standard deviation of the mean. Percent volume differences were evaluated by unpaired (two sample) Student’s T-test.

To calculate the distance between two probes in three dimension, the xyz coordinate of the centroid of each YAC probe signals were determined and measured using Slidebook. The distance between the two probes was the distance between two separate spots closest to one another. The distances where then analyzed over all nuclei within an experimental set to calculate the final median and interquartile range of the data shown in boxplots. Descriptive statistics (minimum, maximum, and sample size) were also calculated. Sample sizes are listed in each figure. Whiskers shown indicate distribution from minimum to maximum. Probe distance differences were evaluated by unpaired (two sample) Student’s T-test.

## Electronic supplementary material

Additional file 1: Figure S1: DCC depletion and mutants result in changes in the volume of the X chromosome. (A) Adult mutant hermaphrodite intestinal nuclei stained with X-paint FISH (red) to label X chromosome territories and DAPI (blue) to label DNA. Representative stained nuclei of wild type, *dpy-30(y228)*, and *dpy-21(e428)*. Scale bars equal 5 μm. (B) Adult RNAi treated hermaphrodite intestinal nuclei stained with chromosome I paint FISH (red) to label chromosome I territories and DAPI (blue) to label DNA after DCC depletion. Representative stained nuclei after vector RNAi treatment, *dpy-30(RNAi)*, *dpy-21(RNAi)*, and *dpy-27(RNAi)*. Scale bars equal 5 μm. (C) Adult mutant hermaphrodite intestinal nuclei stained with chromosome I paint FISH (red) to label chromosome I territories and DAPI (blue) to label DNA. Representative stained nuclei wild type, *dpy-30(y228)*, and *dpy-21(e428)*. Scale bars equal 5 μm. (D) Quantification of the percentage of nuclear volume occupied by individual X chromosome territories (larger territory arbitrarily designated as X_1_ and the smaller territory as X_2_) in control X_1_ (n = 31), control X_2_ (n = 31), *dpy-27(RNAi)* X_1_ (n = 20), and *dpy-27(RNAi)* X_2_ (n = 20). Error bars indicate standard deviation. Asterisks indicate level of statistical significance by t-test analysis (three asterisks, *P* <0.001). (JPEG 438 KB)

Additional file 2: Figure S2: No changes in X or chromosome I size in condensin I or II depleted animals. (A) Western blot analysis of the depletion in adults after one-generation RNAi feeding (*smc-4*, *hcp-6*, *dpy-27*). Each subunit was successfully depleted. Tubulin is shown as a loading control. (B) Adult RNAi treated hermaphrodite intestinal nuclei stained with X-paint FISH (red) to label X chromosome territories and DAPI (blue) to label DNA. Representative stained nuclei after vector RNAi treatment, *smc-4(RNAi)*, *capg-2(RNAi)*, *hcp-6(RNAi)*, and *dpy-27(RNAi)*. Scale bars equal 5 μm. (C) Adult RNAi treated hermaphrodite intestinal nuclei stained with chromosome I paint FISH (red) to label chromosome I territories and DAPI (blue) to label DNA. Representative stained nuclei after vector RNAi treatment, *smc-4(RNAi)*, *capg-2(RNAi)*, *hcp-6(RNAi)*, and *dpy-27(RNAi)*. Scale bars equal 5 μm. (JPEG 368 KB)

Additional file 3: Figure S3: Diploid condensin I and II depleted nuclei show no change in chromosome volume. (A, B) Adult RNAi treated hermaphrodite diploid nuclei stained with X-paint FISH (red) to label X chromosome territories and DAPI (blue) to label DNA. (A) Representative stained nuclei after vector RNAi treatment, *smc-4(RNAi)*, *capg-2(RNAi)*, *hcp-6(RNAi)*, and *dpy-27(RNAi)*. Scale bars equal 5 μm. (B) Quantification of the percentage of nuclear volume occupied by X in vector RNAi (n = 40), *smc-4(RNAi)* (n = 25), *capg-2(RNAi)* (n = 40), *hcp-6(RNAi)* (n = 40), and *dpy-27(RNAi)* (n = 24). Error bars indicate standard deviation. Asterisks indicate level of statistical significance by t-test analysis (three asterisks, *P* <0.001). (C, D) Adult RNAi treated hermaphrodite diploid nuclei stained with chromosome I paint FISH (red) to label chromosome I territories and DAPI (blue) to label DNA. (C) Representative stained nuclei after vector RNAi treatment, *smc-4(RNAi)*, *capg-2(RNAi)*, *hcp-6(RNAi)*, and *dpy-27(RNAi)*. Scale bars equal 5 μm. (D) Quantification of the percentage of nuclear volume occupied by chromosome I in vector RNAi (n = 30), *smc-4(RNAi)* (n = 25), *capg-2(RNAi)* (n = 40), *hcp-6(RNAi)* (n = 40), and *dpy-27(RNAi)* (n = 24). Error bars indicate standard deviation. (JPEG 500 KB)

Additional file 4: Figure S4: Changes in DCC mediated histone modifiers lead to disrupted X chromosomes but not chromosome I. (A) Adult mutant hermaphrodite intestinal nuclei stained with X paint FISH (red) to label X chromosome territories and DAPI (blue) to label DNA. Representative stained nuclei of wild type, *set-1(tm1821)*, *set-4(n4600)*, and *sir-2.1(ok434)*. Scale bars equal 5 μm. (B) Adult mutant hermaphrodite intestinal nuclei stained with chromosome I paint FISH (red) to label chromosome I territories and DAPI (blue) to label DNA. Representative stained nuclei of wild type, *set-1(tm1821)*, *set-4(n4600)*, and *sir-2.1(ok434)*. Scale bars equal 5 μm. (JPEG 268 KB)

## Abbreviations

DCC: Dosage compensation complex
SMC: Structural maintenance of chromosome
CAP: Chromosome-associated polypeptide
H4K16ac: Histone H4 lysine 16 acetylation
H4K20me1: Histone H4 lysine 20 monomethylation
FISH: Fluorescent *in situ* hybridization
YAC: Yeast artificial chromosome
MSL: Male-specific lethal.
