# Supplementary figures and images for: The C. elegans dosage compensation complex mediates interphase X chromosome compaction
Source: Epigenetics Chromatin. 2014 Oct 27;7:31. doi: 10.1186/1756-8935-7-31 (PMC4232692; doi:10.1186/1756-8935-7-31)

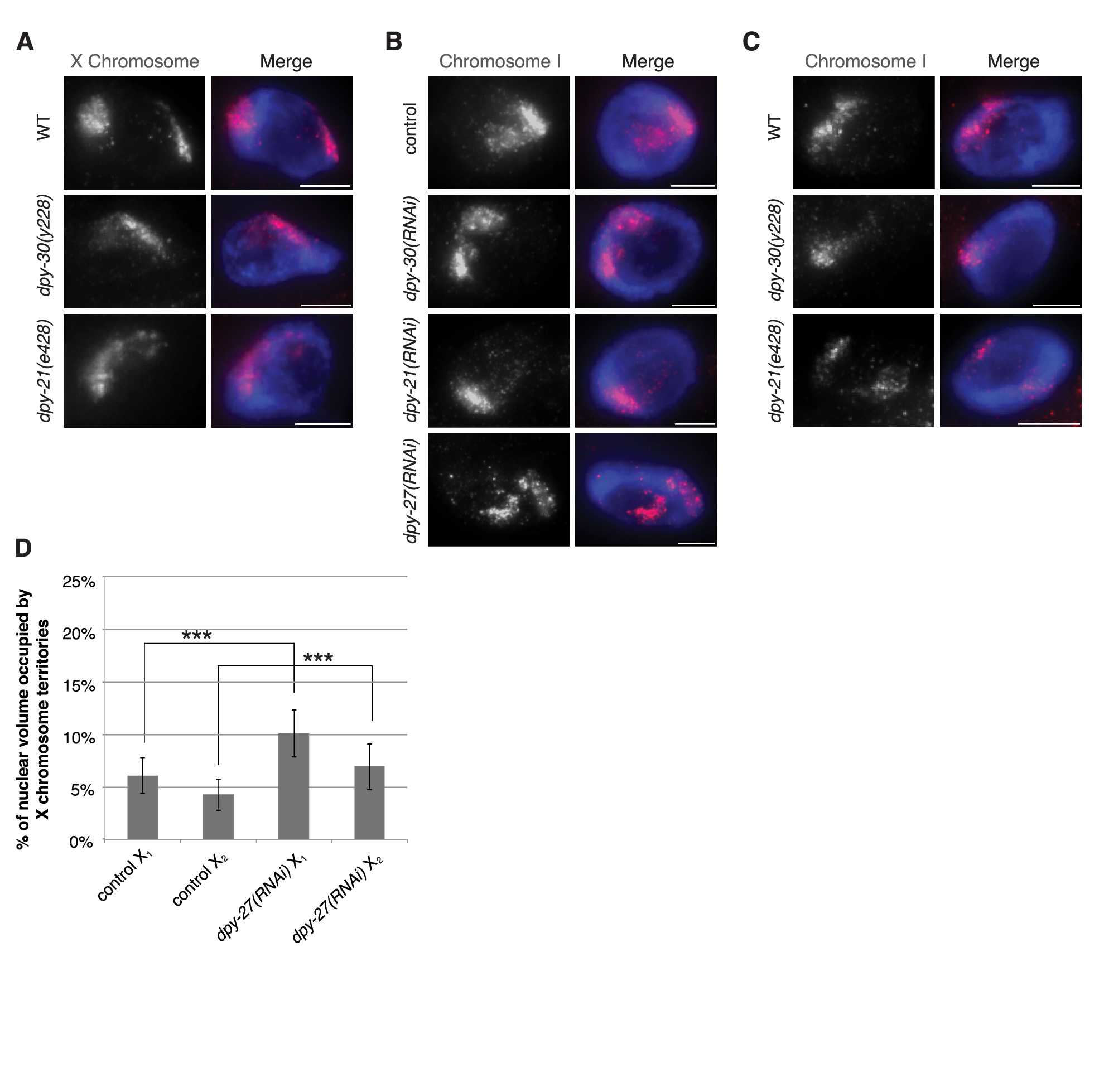

Supplement: Supplementary file 1 — Additional file 1: Figure S1: DCC depletion and mutants result in changes in the volume of the X chromosome. (A) Adult mutant hermaphrodite intestinal nuclei stained with X-paint FISH (red) to label X chromosome territories and DAPI (blue) to label DNA. Representative stained nuclei of wild type, dpy-30(y228), and dpy-21(e428). Scale bars equal 5 μm. (B) Adult RNAi treated hermaphrodite intestinal nuclei stained with chromosome I paint FISH (red) to label chromosome I territories and DAPI (blue) to label DNA after DCC depletion. Representative stained nuclei after vector RNAi treatment, dpy-30(RNAi), dpy-21(RNAi), and dpy-27(RNAi). Scale bars equal 5 μm. (C) Adult mutant hermaphrodite intestinal nuclei stained with chromosome I paint FISH (red) to label chromosome I territories and DAPI (blue) to label DNA. Representative stained nuclei wild type, dpy-30(y228), and dpy-21(e428). Scale bars equal 5 μm. (D) Quantification of the percentage of nuclear volume occupied by individual X chromosome territories (larger territory arbitrarily designated as X1 and the smaller territory as X2) in control X1 (n = 31), control X2 (n = 31), dpy-27(RNAi) X1 (n = 20), and dpy-27(RNAi) X2 (n = 20). Error bars indicate standard deviation. Asterisks indicate level of statistical significance by t-test analysis (three asterisks, P <0.001). (JPEG 438 KB) [file 13072_2014_335_MOESM1_ESM.jpeg]

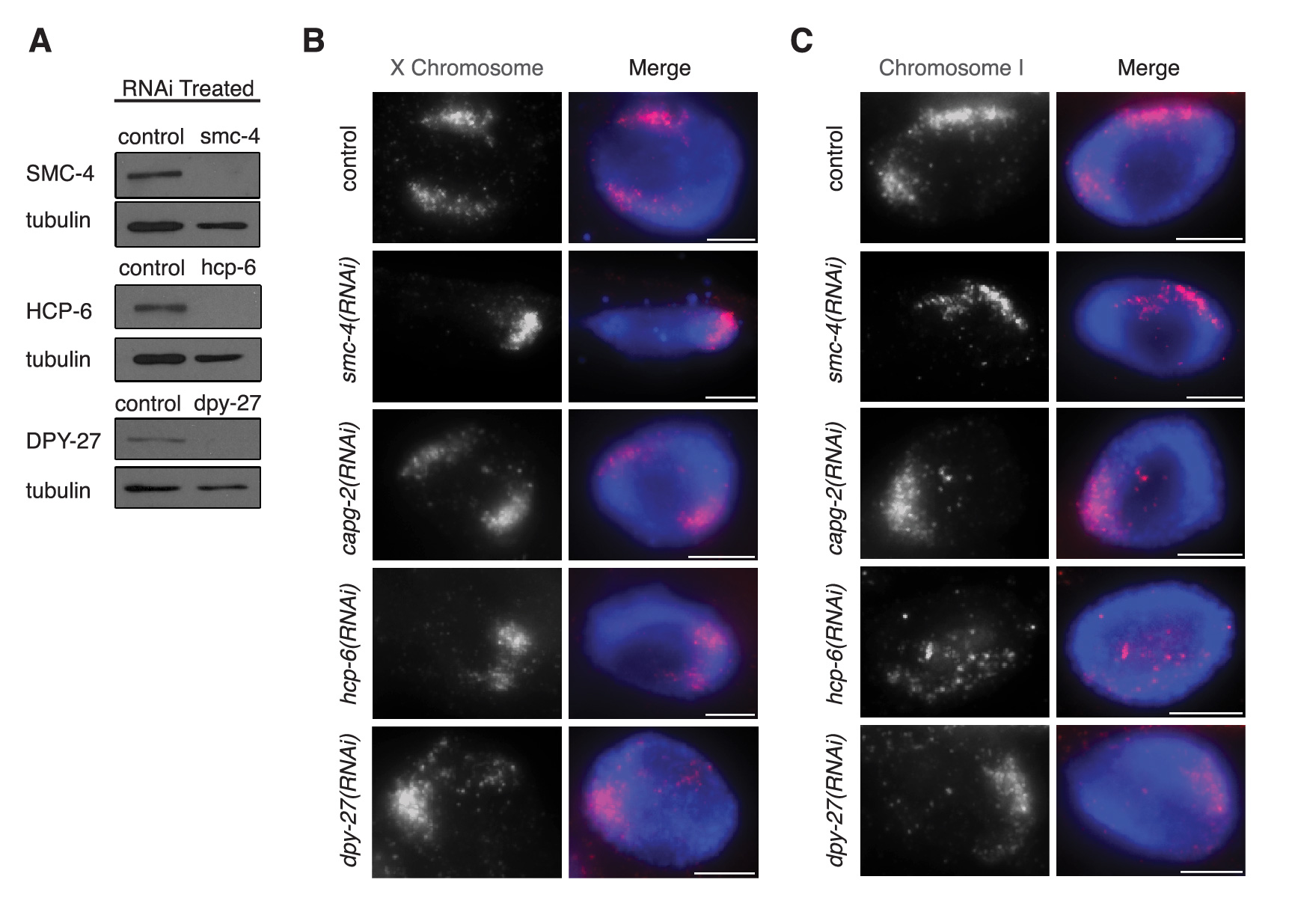

Supplement: Supplementary file 2 — Additional file 2: Figure S2: No changes in X or chromosome I size in condensin I or II depleted animals. (A) Western blot analysis of the depletion in adults after one-generation RNAi feeding (smc-4, hcp-6, dpy-27). Each subunit was successfully depleted. Tubulin is shown as a loading control. (B) Adult RNAi treated hermaphrodite intestinal nuclei stained with X-paint FISH (red) to label X chromosome territories and DAPI (blue) to label DNA. Representative stained nuclei after vector RNAi treatment, smc-4(RNAi), capg-2(RNAi), hcp-6(RNAi), and dpy-27(RNAi). Scale bars equal 5 μm. (C) Adult RNAi treated hermaphrodite intestinal nuclei stained with chromosome I paint FISH (red) to label chromosome I territories and DAPI (blue) to label DNA. Representative stained nuclei after vector RNAi treatment, smc-4(RNAi), capg-2(RNAi), hcp-6(RNAi), and dpy-27(RNAi). Scale bars equal 5 μm. (JPEG 368 KB) [file 13072_2014_335_MOESM2_ESM.jpeg]

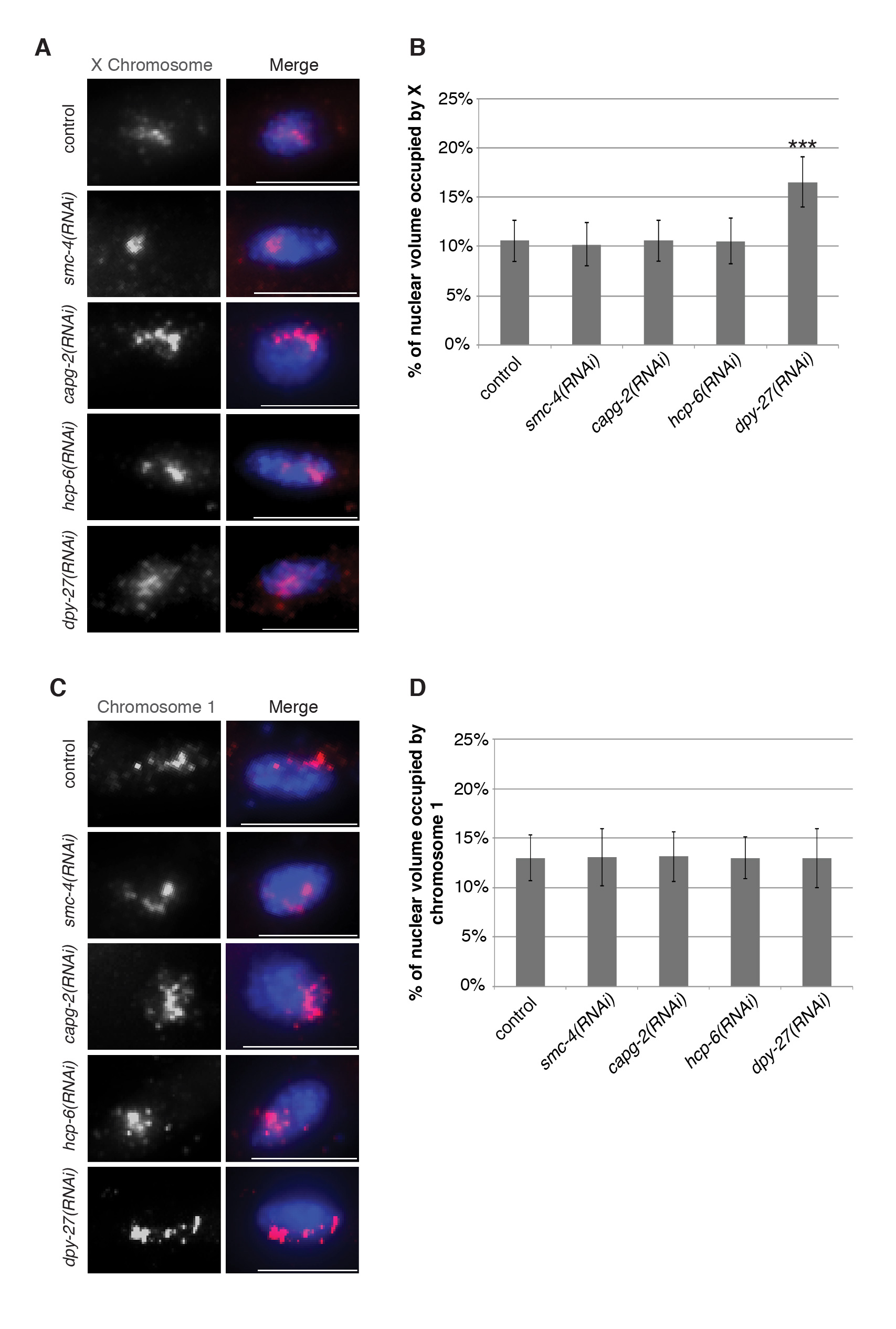

Supplement: Supplementary file 3 — Additional file 3: Figure S3: Diploid condensin I and II depleted nuclei show no change in chromosome volume. (A, B) Adult RNAi treated hermaphrodite diploid nuclei stained with X-paint FISH (red) to label X chromosome territories and DAPI (blue) to label DNA. (A) Representative stained nuclei after vector RNAi treatment, smc-4(RNAi), capg-2(RNAi), hcp-6(RNAi), and dpy-27(RNAi). Scale bars equal 5 μm. (B) Quantification of the percentage of nuclear volume occupied by X in vector RNAi (n = 40), smc-4(RNAi) (n = 25), capg-2(RNAi) (n = 40), hcp-6(RNAi) (n = 40), and dpy-27(RNAi) (n = 24). Error bars indicate standard deviation. Asterisks indicate level of statistical significance by t-test analysis (three asterisks, P <0.001). (C, D) Adult RNAi treated hermaphrodite diploid nuclei stained with chromosome I paint FISH (red) to label chromosome I territories and DAPI (blue) to label DNA. (C) Representative stained nuclei after vector RNAi treatment, smc-4(RNAi), capg-2(RNAi), hcp-6(RNAi), and dpy-27(RNAi). Scale bars equal 5 μm. (D) Quantification of the percentage of nuclear volume occupied by chromosome I in vector RNAi (n = 30), smc-4(RNAi) (n = 25), capg-2(RNAi) (n = 40), hcp-6(RNAi) (n = 40), and dpy-27(RNAi) (n = 24). Error bars indicate standard deviation. (JPEG 500 KB) [file 13072_2014_335_MOESM3_ESM.jpeg]

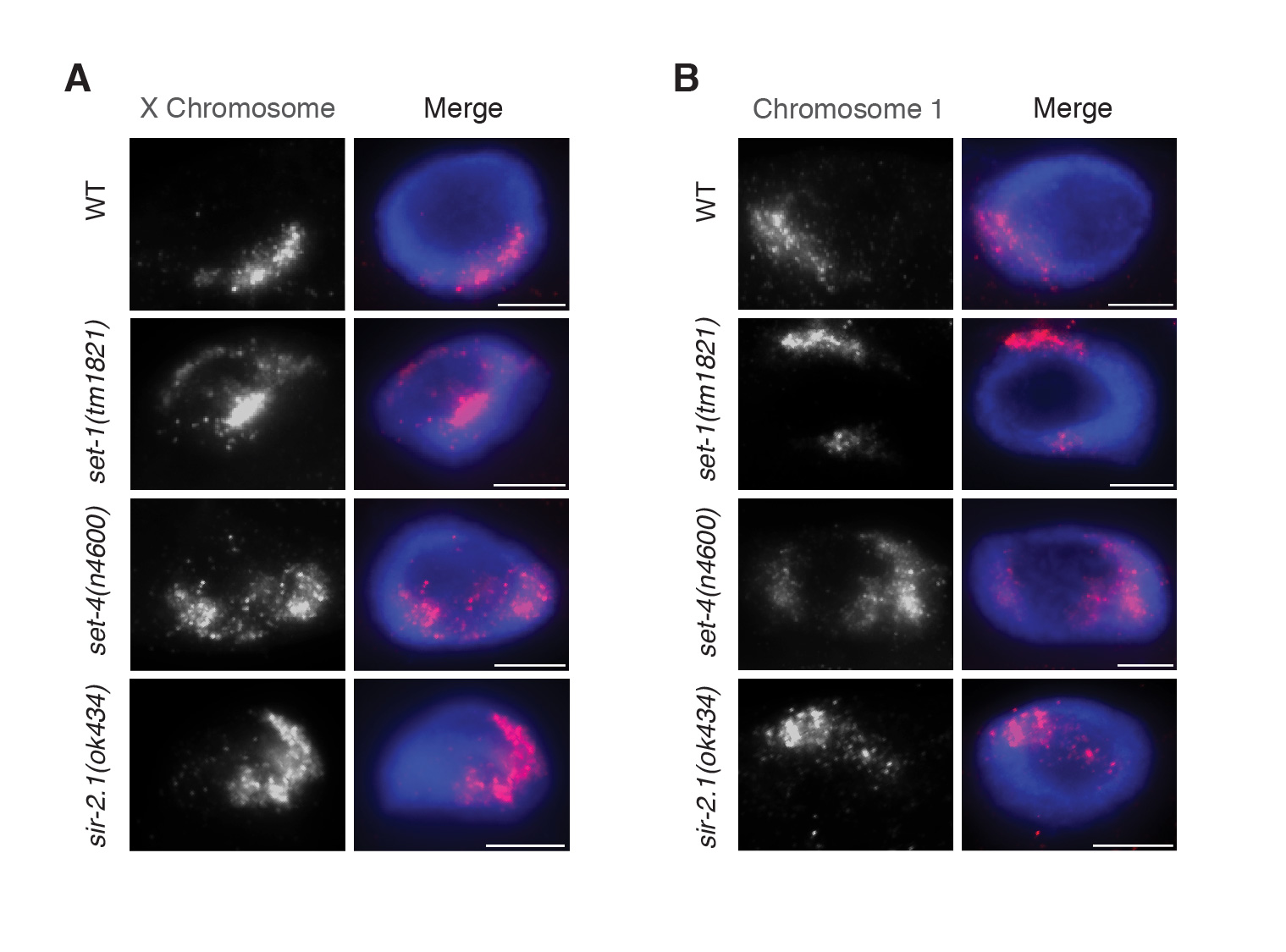

Supplement: Supplementary file 4 — Additional file 4: Figure S4: Changes in DCC mediated histone modifiers lead to disrupted X chromosomes but not chromosome I. (A) Adult mutant hermaphrodite intestinal nuclei stained with X paint FISH (red) to label X chromosome territories and DAPI (blue) to label DNA. Representative stained nuclei of wild type, set-1(tm1821), set-4(n4600), and sir-2.1(ok434). Scale bars equal 5 μm. (B) Adult mutant hermaphrodite intestinal nuclei stained with chromosome I paint FISH (red) to label chromosome I territories and DAPI (blue) to label DNA. Representative stained nuclei of wild type, set-1(tm1821), set-4(n4600), and sir-2.1(ok434). Scale bars equal 5 μm. (JPEG 268 KB) [file 13072_2014_335_MOESM4_ESM.jpeg]
